# Perceptions of ambulance nurses on their knowledge and competence when assessing psychiatric mental illness

**DOI:** 10.1002/nop2.703

**Published:** 2020-11-27

**Authors:** Lizbet Todorova, Anders Johansson, Bodil Ivarsson

**Affiliations:** ^1^ Office of Medical Services Region Skane Malmö Sweden; ^2^ Department of Clinical Science Lund University Lund Sweden; ^3^ Department of Cardiothoracic Surgery IKVL Lund University Lund Sweden

**Keywords:** ambulance services, ambulance specialist nurse, communication, competence, inter‐professional, medical skills, mental illness, mixed methods, psychiatric care, suicide

## Abstract

**Aims and objectives:**

To obtain the current perception of the knowledge and competence of pre‐hospital emergency specialist nurses (ambulance) in attending patients with psychiatric symptoms.

**Background:**

Psychiatric illnesses have increased throughout the population. Consequently, pre‐hospital emergency services frequently attend individuals with suspected or known mental illnesses.

**Design:**

We employed a set of quantitative and qualitative methods to gain a deeper understanding of ambulance nurses' self‐evaluated knowledge.

**Methods:**

Seven ambulance nurses received and completed a survey questionnaire prior commencing employment in November 2019. Then, we conducted interviews to explore ambulance nurses' perceptions of their own knowledge and competence when attending individuals with mental disorders. The surveys were analysed with descriptive statistics, followed by content analysis.

**Results:**

Three topics emerged: the encounter of patients with mental illness; the awareness of lacking knowledge about mental illnesses; and the expectations for future Prehospital Emergency Psychiatric Response Teams. Although ambulance nurses already possessed basic knowledge regarding psychiatric illnesses, it was insufficient, based on their perception of appropriate care. Ambulance nurses considered that combining pre‐hospital and psychiatric expertise in the pre‐hospital emergency unit would increase their in‐depth knowledge about various psychiatric illnesses, the treatment options and the alternatives regarding where to deliver patients for continued care.

**What does this paper contribute to the wider global clinical community?**

Encountering mental illness in a pre‐hospital setting requires teamwork and inter‐disciplinary professional skills, competence and extensive knowledge to instill confidence and provide all‐encompassing medical and nursing care.

## 1 INTRODUCTION

The nurse trained as an ambulance specialist plays an important role in encounters with individuals with mental disorders. They must independently assess the patient's physical and mental conditions; observe, implement and evaluate the patient's care; and treat when necessary. Since 2004, the number of individuals with mental illnesses has increased in Sweden; in particular, the proportion of the population with mental illnesses is slightly higher in southern Sweden than in the rest of the country (Region Skånes Omvärldsanalys, 2017). Increasing emergency calls and ambulance dispatches concerning mental illnesses has placed higher demands on ambulance personnel to perform assessments of acute mental illnesses with appropriate knowledge and ability. Most emergency contacts are related to substance abuse (alcohol and drugs), overdose and behavioural/psychiatric cases, in combination with uncertain information and chest pain, breathing difficulties, unconscious seizures, dizziness or stroke. Among patients with depression or anxiety, less than half had experienced prior treatment (Johansson et al., 2013), despite a previous psychiatric diagnosis and an established open care contact.

Mental illness includes a broad spectrum of signs and symptoms, from mild/intermediate symptoms, like anxiety, depression, suicide attempts, alcohol or drug poisoning, panic disorders, to severe symptoms, like schizophrenia. Worldwide, mental illnesses are becoming a major societal issue, because the prevalence of mental illness is increasing (Mental disorders, 2019; Steel et al., 2014), in both mild and severe forms. Among individuals with mental illnesses, three age groups are particularly vulnerable in different ways: children, young people and older individuals (Holzer et al., 2012; Mental disorders, 2019).

Around the world, the number of incoming calls to emergency medical services that involve psychiatric disorders has significantly increased (Duncan et al., 2019; Prener & Lincoln, 2015; Roggenkamp et al., 2019). Individuals with mental illnesses are probably in greater need of somatic care than many other groups. Emergency and secondary care consultations and unplanned hospital admissions are common, particularly when a patient has a combination of mental and physical conditions (Duncan et al., 2019).

Prolonged mental illness can also cause somatic disorders, such as headaches, gastrointestinal problems or neurological and nervous tension. Moreover, mental illnesses might be associated with cancer, diabetes and heart disease (Byles et al., 2014; Holden et al., 2010; Wang et al., 2007). In Sweden, patients with a combination of severe mental illness and somatic disease are at increased risk of mortality, compared with individuals without severe mental illnesses (Lundin et al., 2016). Previously, a qualitative study explored the barriers and facilitators perceived by patients with severe illness, their relatives and clinicians, which represented primary and somatic health care in Sweden. That study found that healthcare organizational structures and systems failed to facilitate cooperation between different departments (Björk Brämberg et al., 2018). In the current mental healthcare system, both psychiatric and physical healthcare providers must broaden their treatment paradigms to address the whole person and to bridge the existing gap between mental and physical healthcare endeavours (Vreeland, 2007).

In addition to the development of advanced care in pre‐hospital settings, the advent of a pre‐hospital emergency specialist nurse (a registered nurse with one year of additional training in emergency care; here, referred to as an ambulance nurse) is required for all ambulances in the County of Skåne. This requirement represents the highest level of medical competence among the ambulance services in Sweden (Lindström et al., 2015; Suserud, 2005). To ensure good quality care, nurses must be highly competent and trained in the appropriate attitudes in their approaches to patients, mental health work and themselves, as a healthcare provider. In general, ambulance nurses must have the appropriate knowledge and competence to ensure that both somatic and psychiatric illnesses are adequately recognized and treated. Several previous studies indicated that the scope of paramedic practices varies, depending on their education, degree and work experience. These characteristics influence the nature and extent of the care they provide (McCann et al., 2018; Roberts & Henderson, 2009; Sjolin et al., 2015). The two major obstacles that prevent patients with mental illnesses from receiving the appropriate care are negative attitudes and a stigma towards mental illnesses. Recent studies have shown that, compared with nurses in psychiatric care (Chambers et al., 2009), nurses working in somatic care have a higher degree of negative attitudes towards individuals with severe mental illnesses. In general, nursing staff attitudes are comparable, in several respects, to public perceptions about mental illnesses (Björkman et al., 2008).

Because mental health disorders are becoming a nationwide healthcare concern, alternative approaches have been introduced. Evaluations of these approaches showed that a first‐line response that included combined psychiatric and somatic expertise was beneficial in providing advanced medical assessments, when attending individuals with severe mental health issues, behavioural crises or suicidal tendencies (Boscarato et al., 2014; Bouveng et al., 2017; Evangelista et al., 2016; Huppert & Griffiths, 2015; Lindström et al., 2020; Nordentoft et al., 2002; Watson et al., 2008). Therefore, the Board of Health in Region of Skåne decided to initiate, for a limited period of time, a project that included an additional pre‐hospital emergency psychiatric unit in the southeast part of Sweden. This unit included ambulance nurses accompanied by a specialist nurse trained in psychiatry, and it was dispatched by emergency call operators, when they received a call and identified a mental health‐related crisis that required the pre‐hospital emergency psychiatric unit.

As a first step in evaluating whether a pre‐hospital psychiatric unit might lead to increased knowledge and competence in pre‐hospital ambulance care, the present study aimed to determine the current perceptions of ambulance nurses' knowledge and competence, when attending patients with psychiatric symptoms. We also investigated ambulance nurses' expectations of whether collaborations with a psychiatric nurse, in the future, should be included in the pre‐hospital emergency psychiatric unit in southern Sweden.

## 2 METHODS

### 2.1 Research design

This study applied a set of quantitative and qualitative methods design. First, we conducted a quantitative survey, with a questionnaire that contained fixed, multiple‐choice options. Second, we conducted qualitative interviews to provide a more complete picture of the context.

### 2.2 Participants and procedure

Immediately after starting the pilot project, ambulance nurses working in the Ambulance Service where the pre‐hospital emergency psychiatric unit was to be placed had the opportunity to apply and participate in the project prior employment. Those ambulance nurses that were recruited to pre‐hospital emergency psychiatric unit and showed interest in participating in the project were contacted and information about the study was given early in the project.

Seven senior ambulance nurses (all with ≥5 years professional nurse experience) participated in the study after providing written informed consent. The questionnaire with fixed, multiple‐choice options (Svensson & Hansson, 2014) was completed in September and October 2019. Immediately after completing the questionnaire, all seven ambulance nurses underwent individual qualitative interviews. All interviews were recorded, and they lasted 27–47 min.

### 2.3 Ethics approval and consent to participate

Ethics approval was obtained from the Swedish Ethical Review Authority [Dnr: 2019‐04040]. The study protocol was approved by the department head. All respondents were informed that their data would remain confidential and that they could withdraw participation at any time without explanation. All data were stored securely and access was permitted only to the research team.

### 2.4 Data analyses

#### 2.4.1 Quantitative section

The questionnaire was tested in a pilot study with three senior ambulance nurses that were not involved in the main study. Based on their responses, no changes were made. All variables are expressed as absolute and relative frequencies (*n*, %), the mean ± *SD* or the median and interquartile range, when appropriate (Altman, 1991). The question‐index value (Q‐IV, range: 0–1.0) was defined as a summary of the proportion of *positive* responses (%), where a *positive* response was defined as a rating from “*To a quite large extent*” to “*To a very large extent*.” All analyses were performed with SPSS^®^ version 24.0 (SPSS Inc., Chicago IL),

#### 2.4.2 Qualitative section

Qualitative data were analysed with conventional content analysis, inspired by Hsieh and Shannon (Hsieh & Shannon, 2005) and following consolidated criteria for reporting qualitative research, (COREQ) (Tong et al., 2007). The interviews focused on the ambulance nurses' experiences and expectations of care for patients with mental illnesses. The interview questions were tested in one interview (excluded from the analysis) to check the relevance and clarity. The interviews commenced with two open questions to the respondents: “*Can you tell me about your experiences of caring for patients with mental illnesses in an ambulance care setting*?” and “*Can you tell me about your expectations about the future prehospital emergency psychiatric unit*?”. These questions were followed‐up with questions, such as “*Can you tell me more?*” and “*What did you feel?*” for clarification and to encourage more conversation.

The interviews were audio‐recorded, transcribed verbatim and then read carefully several times by the authors (BI and AJ). We created a good overview of the material and identified meaningful experiences that corresponded to the study aim. During the structural phase, meaningful text units were identified that corresponded to the purpose of the study. These units were copied from the text, and each meaningful object received a code that highlighted what the text contained. Then, we sorted the code words into main categories and subcategories. Throughout the analytical process, all authors (BI, AJ and LT) repeatedly discussed the categories, until we reached an agreement. The final results are presented in three main categories and 13 subcategories (Table 3). Quotes from the interviews were used to underpin and reinforce the results.

## 3 RESULTS

### 3.1 Descriptive findings

Of the seven ambulance nurse respondents, two were women and five were men (*n* = 2 women vs. *N* = 5 men) had a mean age of 45 years (*SD* 7, *range:* 34–56 years). All seven respondents had worked as a ambulance nurse for an average of 13 years (*SD* 10, *range:* 2–28 years) and are thus referred as senior ambulance nurses. Quotations from all seven research participants (Respondents, R) are included and referred in the text R1‐R7.

### 3.2 Quantitative findings

Table 1 shows the multiple‐choice responses and the Q‐IVs. Table 2 shows the appellations associated with the Q‐IVs.

**TABLE 1:** Quantitative questionnaire responses and the question‐index value (Q‐IV, range: 0–1.0)^a^

Questions	Multiple‐choice responses (%)					*Q‐IV*
	Not at all	To some extent	To a quite large extent	To a large extent	To a very large extent	
Q1. I feel safe with a person who does not seem to feel good mentally	0	0	43	43	14	1.0
Q2. I am comfortable listening and talking to a person about their mental health problems	0	14	14	43	29	0.86
Q3. I know what to listen to and pay attention to when I talk to someone who is down and sad	0	57	43	0	0	0.43
Q4. I dare to ask if whether a person has thoughts of taking their own life/committing suicide	0	0	14	43	43	1.0
Q5. I can give people who are mentally ill information about what types of effective help are available	0	71	14	14	0	0.28
Q6. I can inform a person where to turn for the right kind of help	0	71	29	0	0	0.29
Q7. I recognize signs that a person has a mental illness	0	0	43	57	0	1.0
Q8. I can judge the seriousness of a situation when a person is in a severe mental crisis	0	14	57	29	0	0.86
Q9. I can suggest things/actions that can comfort a person with a mental illness	0	86	14	0	0	0.14

^a^ The Q‐IV is the fraction of positive perceptions; a positive perception was defined as any response from “*To a quite large extent*” to “*To a very large extent*”.

**TABLE 2:** The question‐index values (Q‐IV, range: 0–1.0)^a^ and their perception appellations

Question	Mean Q‐IV	Perception Appellations^b^
Q1. I feel safe with a person who does not seem to feel good mentally	1.0	*Very good perception*
Q2. I am comfortable listening and talking to a person about their mental health problems	0.86	*Very good perception*
Q3. I know what to listen to and pay attention to when I talk to someone who is down and sad	0.43	*Fairly good perception*
I dare to ask whether a person has own life/committing suicide	1.0	*Very good perception*
I can give people who are mentally ill information about what types of effective help are available	0.28	*Bad Poor perception*
I can inform a person where to go for the right kind of help	0.29	*Bad Poor perception*
Q7. I recognize signs that a person has a mental illness	1.0	*Very good perception*
Q8. I can judge the seriousness of a situation when a person is in a severe mental crisis	0.86	*Very good perception*
Q9. I can suggest things/actions that can comfort a person with a mental illness	0.14	*Bad Poor perception*
***Overall perception*** ^c^	0.65	*Good perception*
(mean Q‐IV)		

^a^ The Q‐IV is the fraction of positive perceptions; a positive perception was defined as any response from “*To a quite large extent*” to “*To a very large extent*” (see Table 1).

^b^ Appellations were assigned according to the Q‐IV, as follows: <0.20 = No or very poor perception; 0.21–0.40 = Poor perception; 0.41–0.60 = *Fairly good* perception; 0.61–0.80 = *Good* perception; and 0.81–1.00 = *Very good* perception.

^c^ The mean Q‐IV is the *Overall* perception.

The Q‐IVs for questions 1, 2, 4, 7 and 8 suggested that ambulance nurses had *Very good perceptions* (Q‐IVs = 0.68–1.0) of how to treat individuals with mental illnesses. The results demonstrated that the ambulance nurses felt *safe* (Q1) and *comfortable listening and talking* (Q2) with individuals that had mental health problems. The ambulance nurses also *dared to ask whether a person had thoughts of taking their life/committing suicide* (Q4), they recognized *signs that a person had a mental illness* (Q7), and they seemed to be comfortable in judging *the seriousness of a situation, when a person was in a severe mental crisis* (Q8). However, it was clear that the ambulance nurses had poor perceptions of how to *inform individuals about what kinds of effective help are available* (Q5), *where to go for the right kind of help* (Q6) and how to perform *things/actions that might comfort a person with a mental illness* (Q9) (Table 1).

Based on the mean Q‐IV (0.65), the *Overall perception* of ambulance nurses in handling patients with mental illnesses corresponded to an appellation of *Good perception* (Table 2).

### 3.3 Qualitative findings

The overall message that emerged from this study was that encountering mental illness in a pre‐hospital setting required inter‐disciplinary professional skills, competence and extensive knowledge to instill confidence and provide all‐encompassing medical and nursing care. This message was interpreted from the experiences described by the seven senior ambulance nurses in individual interviews. These experiences were categorized as: encounters with patients that had mental illnesses; an awareness that knowledge about mental illnesses was lacking; and the expectations for a future Prehospital Emergency Psychiatric Response Team. These main categories were populated with thirteen subcategories (Table 3).

**TABLE 3:** Quality analysis of ambulance nurse experiences and expectations when attending patients with psychiatric symptoms

Main categories	Subcategories
*Encounters with patients that had mental illnesses*	Complex encounters
	The importance of adequate information
	Difficulties with adequate diagnoses
	Significance of building trust and confidence
	Strategies for personal security
	Involvement of relatives
	Culture and language barriers
	Deficient alternatives for higher levels of care
*Awareness that knowledge about mental illnesses was lacking*	The necessity of specific knowledge and clinical skills
	Existing care‐programmes provided limited guidance for individual needs
*Expectations for a future Prehospital Emergency Psychiatric Response Team*	Increased knowledge and collaboration
	Enhanced care for patients
	Belief in the positive effects of collaborating with other actors

#### 3.3.1 Encountering patients with mental illnesses

##### Complex encounters

The ambulance nurses expressed that ambulance health care was the most multifaceted context to work in and that alerts involving mental illness were common. Although most respondents had a specialist degree in ambulance care, they described a lack of in‐depth knowledge about mental disorders, probably because the ambulance care curriculum had focused on the assessment and treatment of medical diseases. The respondents also mentioned that patients with mental illnesses often had other medical diseases and/or had been exposed to different traumas. Consequently, ambulance nurses often concentrated their assessments and treatments on the medical disorders, without paying close attention to a contemporary mental illness. “*So the physical problems are what we deal with—not with the psychiatric illness. Right or wrong, that's how it is*.” (R2).

##### The importance of adequate information

To date, the ambulance organization has no access to patient records. Most ambulance nurses wish that they could obtain more background data to support their assessments of the situations. Furthermore, background information was considered important in managing situations of potential violence. However, at the same time, the ambulance nurses expressed concerns that more background data could lead to negative preconceptions about some patients. *“Sometimes, according to the encounter with the patients, I think it's good to be an ‘blank slate’, where you don't know anything. But sometimes, I think some patients would have wished that we had known their history so that they would not have to repeat it [the history]*” (R5).

##### Difficulties with adequate diagnoses

It often happened that the ambulance nurse received a traditional medical diagnosis from the dispatch centre or from a patient or a relative on arrival at the scene, when in fact, the incident involved a patient with a chronic psychiatric illness. Making a secondary diagnosis in the context of the mental illness spectrum was often based on an “exclusion method.” Somatic diagnoses, such as myocardial infarction, respiratory distress or abdominal pain, which might be mistaken as a mental illness, could often be excluded based on the patient's medical history and the actual vital assessment parameters. The ambulance nurses stated that some patients had aversive responses to physical contact, which made it difficult for the ambulance nurse to examine the patient to determine whether an injury had occurred. In addition to questioning the patient about drugs and drug intake, the ambulance nurse observed the scene to detect potential drug accessories, to facilitate a differential diagnosis. Therefore, the nurses felt that it was extremely important to take the time to ask questions about the psychological mood and the psychosocial situation. “*If all the vital parameters are green [within normal limits] and the patient still feels that something is wrong, you can start to think about other signs: it may be how they express themselves, the tone of voice, their body posture, or how they relate to us and to the environment*” (R1).

##### Significance of building trust and confidence

It became clear that the ambulance nurses always tried to understand the patient and the situation in concrete conversations with patients with mental illnesses. It often emerged that it was important to communicate calmly and objectively, before proposing treatment and/or suggesting actions. Thus, the ambulance nurse did not promise anything that might not be fulfilled. The ambulance nurses’ perceptions were that a conversation should “invite the patient to enter into a dialogue,” and the patient should not perceive the dialogue as imperative. Therefore, the ambulance nurses stated the meeting should be infused with the attitude of “meeting the person” and management should be based on the patient's needs. Through their experiences, the ambulance nurses had learned that it was best to always ask straightforward questions. This approach was also considered very important for patients with suicidal ideations. At the same time, the ambulance nurses were always aware of and prepared for, the possibility that the situation could continuously change. “*It's a patient group that is quite marginalized … I don't feel I can do as much for them as I can do for patients with a somatic ailment*” (R6).

##### Strategies for personal security

In alerts with elements of mental illness, the ambulance nurses believed that attending to their own security concerns came first. Ambulance nurses stated that the ambulance colleague was very important in these situations. Often, there was a strategy for each meeting. For example, the ambulance nurse would first scan an apartment and the environment to take in its appearance and to determine the presence of knives or other dangerous objects, empty drug packaging, liquor bottles and/or whether children were involved in the situation. The crew often split their focus in these situations. One crew member would focus on the patient, and the other crew member would more closely monitor the entire situation. In patient‐centred work, the ambulance nurses elucidated some physical strategies that they used to protect themselves from danger. This strategy included precautions that they took when they entered an apartment; for example, they placed the equipment between themselves and the patient and they always had a retreat strategy to get out of the situation. “*I do not distrust people to a great extent, but in these situations, with patients that have mental illnesses, my colleague and I are probably more cautious*” (R3).

##### Involvement of relatives

Many times, in situations involving a mental illness, the relatives had to make the emergency call and sometimes, the patients were not aware of the arrival of the ambulance crew. The respondents stated that they sometimes split up the patient and the relatives to get independent views of the situation. This manner of handling the situation was considered very important in situations involving aggressive behaviours. The ambulance nurses also stated that, sometimes, their role was to provide emotional support to the relatives. “*Often relatives have called for an ambulance. This often happens when things get crazy and they [the patients] start screaming and breaking things*” (R2).

##### Culture and language barriers

The ambulance nurses described dilemmas that they faced when the patient with a mental illness was from another culture and/or had language difficulties. The respondents felt that patients from some cultures appeared to be more stigmatized by mental illness than individuals from Sweden; thus, in the first contact with the emergency centre, they often described the condition as a physical illness. Another problem was that the ambulance staff did not usually have access to a professional interpreter. To manage these situations, they often recruited close relatives that could participate in and interpret the conversation, sometimes even on the telephone. The ambulance nurses were aware that there could be emotional involvement that might have a negative impact on the translation. However, they observed the patient's body language and sometimes used the online translation service—*Google translate*. The ambulance nurses felt that this was an accessible approach for understanding the patients' situation. “*If there is a relative that can translate into Swedish, then it is alright. Otherwise, we have problems, because we do not know what they are saying*” (R6).

##### Deficient alternatives for higher levels of care

It also happened that, after a dialogue with the Regional Medical Support, patients were left at home and invited to contact their “own‐physician” or a medical centre. The ambulance nurses stated that they did not have many choices, when it came to assessing the right level of care for these patients; that was something they felt they were missing. The existing choices were either to transfer the patient to the emergency department, when a somatic injury was present or when tablets or drugs were involved, or to transfer the patient to a traditional psychiatric emergency centre for adults or children or a specific drug abuse centre. In addition to the fact that they sometimes felt resigned to the use of alternative treatment authorities, they stated that stigmatization was likely to be prevalent in a somatic emergency department. They linked this stigmatization to the fact that the medical staff [in‐hospital] routinely looked at the records and concluded that the patient, once again, needed care for the same reasons. Therefore, many respondents felt that no authority took full responsibility for these patients. “*In general, I think we can do a little bit better and take each new visit as a new visit and treat every patient in the same way. But it becomes difficult when you meet them 15 times … My opinion is that everyone should be treated in the same way, at all times*” (R4).

#### 3.3.2 Awareness that knowledge about mental illnesses was lacking

##### The necessity of specific knowledge and clinical skills

Many patients had isolated mental illnesses; thus, the respondents were convinced that they needed education/training in this area. It also emerged that the ambulance healthcare staff generally had little contact with psychiatry departments to exchange knowledge and experiences. The respondents also specifically stated that the curriculum for the specialist programme in ambulance care should include more education in the field of mental illness. To “read a book and then take a test” was considered insufficient. The respondents called for practical training in psychiatry departments to gain more understanding and knowledge about psychiatric illnesses. “*But I think it is absolutely necessary to have more education, because mental illnesses are encountered in emergency care and ambulance care to such an extent that we also need practical training*” (R7).

##### Existing care‐programmes provided limited guidance for individual needs

The respondents stated that the local psychiatry guidelines for the ambulance care service could be of some help. It was good that they had something to “fall back” on. However, the respondents stated that a written care programme was only partially useful, because in reality, meeting with a patient often imposed other demands on the care provider. However, some recommendations that related to the overall encounter were found to be helpful. Therefore, the care programme was perceived as a good supplement, because it guided the ambulance nurses in the assessments. “*For all our programs, you read it, but you will not remember all the details; however, it provides a guide anyway*” (R6).

#### 3.3.3 Expectations for a future Prehospital Emergency Psychiatric Response team

##### Increased knowledge and collaboration

The respondents stated that they had limited competencies in the context of psychiatric disorders, and they expected their competencies to be developed in conjunction with the introduction of the emergency psychiatric response team project. They expected that collaborating with psychiatric specialist nurses would increase their own general knowledge about the various psychiatric illnesses, the treatment options and the alternatives for selecting where to deliver patients for continued care. The collaboration was expected to facilitate the development of skills in communication and supervision. Over time, the ambulance nurses expected to acquire extensive knowledge in the context of mental illnesses, which they could share with their colleagues in the ambulance care team; thus, they could indirectly increase the overall competencies in their profession. “*I expect to learn something more about psychiatry, more in‐depth. So that I feel more equipped to take care of these patients … and then I can teach my colleagues*” (R7).

##### Enhanced care for patients

The ambulance nurses were convinced that patients would gain a better understanding of their own health condition through the specific psychiatric knowledge and skills provided by the *Prehospital Emergency Psychiatric Response* team. Through this project, the ambulance team would have access to patient medical records. The ambulance nurses expected that this access would facilitate patient management, because treatments could be based on previous treatment alternatives, for patients that had established healthcare contacts for their mental illness. Accordingly, ambulance nurses expected that fewer patients would have to be transported to the psychiatric emergency department and that the ambulance nurses could more easily motivate their decisions for eventual relatives. The ambulance nurses were also convinced that the new psychiatric team vehicle, which would be equipped with typical seats instead of a stretcher, would improve the conversational climate. “*Patients with mental illnesses will not have to lie down when transported … it is not the body that is sick. In the psychiatric vehicles … they can sit up and talk with us in a different way*” (R5).

##### Belief in the positive effects of collaborating with other actors

The ambulance nurses stated expectations that the psychiatric attendance might result in fewer compulsory transports for patients that need psychiatric care. They also believed that the psychiatric specialist nurse, which would be in direct contact with physicians in the psychiatry department, could provide more confidence, security and adequate information to the patient. In addition, they expected that the ambulance care service would need fewer collaborations with police units and other physicians, in cases that required “written medical certificates”; moreover, they expected that “forced entries” would be required less frequently. On the other hand, the ambulance nurses doubted that the psychiatric team could avoid transports to the medical emergency department, because patients under the influence of drug and/or alcohol are often denied access to drug addiction units and/or the psychiatric department. The ambulance nurses expressed some frustration over current management practices, because the respondents frequently found that patients departed the emergency department before they had received adequate care for their mental illness. “*Hopefully, the patients will feel more calm and secure when they know that they will get help from the psychiatry unit, which probably will also lift some of the burden off both the police force and … other authorities'… No one wants the police force to take part, because you often find it embarrassing*” (R3).

## 4 DISCUSSION

Through our mixed methods of quantitative and qualitative approaches, seven senior ambulance nurses provided important insights, through self‐assessments and the expression of personal perceptions, regarding their care for people with mental illnesses. Although the ambulance nurses felt safe and comfortable listening and talking to a person about their mental health problems or asking about thoughts of committing suicide, these nurses seemed to be less comfortable in judging the seriousness of a situation, when a person was experiencing a severe mental crisis. Moreover, most respondents expressed inadequate (poor) perceptions in providing patients about what effective help is available (Q5), knowing where to turn for the right kind of help (Q6) and providing or recommending self‐care for the patient. This feeling of inadequacy was probably one of the reasons that ambulance nurses described encounters with mental illnesses as a complex experience. Most often, they focused on the medical condition and paid less attention to the mental health of the patient. Despite having completed a specialist degree in ambulance care, the respondents felt that they lacked in‐depth knowledge about various mental disorders and comorbidities, particularly when patients had been exposed to different traumas. Several studies have shown that people with mental disorders, besides having more health problems and less adherence to medications, also tend to be at higher risk of complications after severe trauma, such as self‐inflicted injury, attempted suicide or penetrating trauma, where one‐third of major trauma patients had used alcohol, drugs or a medication overdose at the time of admission (Byles et al., 2014; Clous et al., 2017; Dekker et al., 2020; Holden et al., 2010; Lundin et al., 2016; Søvsø et al., 2019; Wang et al., 2007).

An increase in the use of hospital and pre‐hospital emergency services, due to acute recreational drug and alcohol toxicity (Archer et al., 2013), is often associated with an increased risk of suicide. In Sweden, about 25% of those who committed suicide were affected by alcohol when they died (Lundholm et al., 2013). Although it is known that repeated suicide attempts are common during the first and second years after a first suicide attempt, we lack efficient suicide prevention strategies (Zalsman et al., 2016). The risk of suicide is very high among young men that have experienced a few years of schizophrenic illness and that have concomitant social problems, such as difficulties with family, education or work (Haglund et al., 2019).

Our respondents felt that it was extremely important to take the time to ask questions about the psychological mood and the psychosocial situation. They stated that meetings should be infused with the attitude of “meeting the person,” and management should be based on the patient's needs. Through experience, they learned that it was best to always ask straightforward questions, particularly with patients that had suicidal ideations. The meeting between the ambulance nurse and the patient can play a major role in preventing suicide at a later date, because a suicide attempt is considered more of a risk factor for suicide completion than previously thought (Bostwick et al., 2016).

A study by Holmberg and Fagerberg (2010) investigated the responsibilities of ambulance nurses to the patient in a pre‐hospital setting. They showed that it was essential for the ambulance nurse to prepare and create conditions that facilitated nursing care, to be there for the patient and to provide comfort and care to those close to the patient and significant others (Holmberg & Fagerberg, 2010). Working in a team, the strategy of assigning the ambulance nurse to focus on the patient and assigning the other ambulance crew members to monitor the surrounding environment benefitted the team's own security and promoted the holistic perspective of care. In each encounter, the patient's and their significant others' lifestyle was an essential factor that influenced how the nurse accomplished the care. A study by Sebergsen et al. (2016) showed that confirming mental healthcare acts seemed to comfort individuals in a step‐wise manner during a psychotic illness (Sebergsen et al., 2016). The ambulance nurse's efforts to adjust the care according to the changing care needs of an individual with a psychotic illness was shown to confirm the need for care that helped the individual feel better, which in turn, might have enhanced its health and increased the quality of acute psychiatric care.

In the present study, some of the ambulance nurses stated that they occasionally preferred the lack of access to patient records, because it helped them encounter the patient with no pre‐determined assumptions and thus, perform the correct assessments. However, in other situations, they felt that a previous medical history might have been helpful, from the patient's point of view. Other barriers to adequate care were the inability to speak the patient's language and understand the patient's culture. Dedicated cross‐cultural training programmes might increase care skills and promote equal care, regardless of ethnicity (Vicente et al., 2019). Since 2011, the influx of refugees to Sweden has increased. These refugees face a higher risk of psychiatric disorders, including post‐traumatic stress disorder, psychosis and schizophrenia, compared with other, non‐refugee migrants in the Swedish population (Hollander et al., 2016; Tinghög et al., 2017). In this context, ambulance nurses have experienced an acute awareness of their lack of knowledge on specific types of mental disorders, despite the local psychiatry guidance programme.

Many patients with mental disorders are repeatedly in need of emergency care; however, as our respondents stressed, in certain cases, the ambulance nurses did not have many choices of places that could offer the appropriate care. In addition to the close collaboration with their colleagues in the ambulance team, ambulance nurses felt that collaborations with other relevant healthcare institutions were even more important, when handing over the patient. All our ambulance nurses reported a feeling of resignation, due to the lack of alternatives for further levels of care. Moreover, these feelings had a negative effect on their ability to take responsibility for the patients. Indeed, a reorganization of the present healthcare organizational structures and systems is required to facilitate cooperation between different departments and to provide the appropriate level and place of care for the patient (Björk Brämberg et al., 2018; Vreeland, 2007). Otherwise, there is a risk that patients with mental and physical disorders might be repeatedly refused admission to either a psychiatric or medical ward, because patients under the influence of drugs and/or alcohol are often denied access to drug addiction units and/or the psychiatric department. Moreover, even when they are eventually admitted, they may be discharged or transferred early to another ward. Indeed, ambulance nurses indicated that stigmatization against these patients was prevalent among somatic emergency departments. This finding pointed out the lack of equity between psychiatric and somatic wards.

In the first‐year follow‐up of the Psychiatric Emergency Response Team in Stockholm County, Sweden (Bouveng et al., 2017), it was found that patients with psychiatric disorders received high‐quality pre‐hospital assessments, which reduced the workload of the police and ambulance services. At the same time, this team contributed to reducing the stigmatization of psychiatric illnesses. Thus, psychiatric ambulances are important in society, because they contribute to the goal of equating mental illnesses with somatic diseases.

Our respondents described their expectations for the upcoming project that would combine pre‐hospital and psychiatric expertise in the pre‐hospital emergency unit in the county of Skåne. They expected that this project would increase their general and in‐depth knowledge about various psychiatric illnesses, the treatment options and the various places for patients to receive continued care. All of our respondents were convinced that the patients would have more confidence in pre‐hospital care, due to the positive effects of inter‐professional collaborations.

## 5 STUDY LIMITATIONS

One potential limitation in our study is the limited number responders of ambulance nurses being employed in the pilot project with additional pre‐hospital emergency psychiatric unit. At the same time, all seven ambulance nurses had ≥5 years professional nurse experience and the individual interviews were considered rich in content. An important consideration in this context is what the experience of novice ambulance nurses would have offered as compared with their senior counterparts and whether unequal representation of gender may have affect the results. Psychiatric illness is associated with stigma and age and professional experience do have an influence on attitudes to specific mental illnesses. Of importance, frequent contacts with persons with mental illness over time were found related to less negative attitudes and prejudices (Björkman et al., 2008). We believe that our semi‐structured questions and the possibility of adding further information prior closing interview strengthen credibility in our results.

## 6 CONCLUSION AND RELEVANCE TO CLINICAL PRACTICE

We found that there is an urgent need for ambulance nurses to learn more about the onset and symptoms of psychiatric illnesses and the appropriate approach for assessing situations of psychiatric character. It is highly important to adjust the competence of the pre‐hospital care team, on multiple levels. The first level begins with the emergency call; the next levels are patient assessments and triaging by the ambulance crew; and the final level is delivering the patient to the appropriate place for definitive care.

The county of Region Skåne is a dynamic, expanding region with 1.3 million inhabitants. In urgent situations, patients might receive extensive access to specialist expertise, with or without the collaboration of other blue light authorities. Patients with mental illnesses might feel less stigmatized in a first meeting with a healthcare professional, compared with any of the other blue light authorities. An emergency care team with combined psychiatric and ambulance specialist expertise can improve the safety of on‐site patient assessments and refer patients to an appropriate level of care. This could increase patient safety and reduce the number of patients transported to the emergency room. A better understanding of mental health, pre‐hospital care and associated methods and organizations might improve the quality of assessing patients with both mental and somatic health issues.

## CONFLICTS OF INTERESTS

No conflicts of interest, funding or personal relationship have been declared by the authors. Authors confirm agreement with the final statement.

## AUTHORS' CONTRIBUTIONS

**Lizbet Todorova**: Conceptualization, methodology, visualization, writing—original draft and writing—review and editing. **Anders Johansson**: Methodology, formal analysis and writing—review and editing. **Bodil Ivarsson**: Methodology, investigation, formal analysis and writing—review and editing. The manuscript has been approved by all authors.

## Data Availability

The data supporting the findings of this study are available from the corresponding author, upon reasonable request.
